# Genetic liability to multiple factors and uterine leiomyoma risk: a Mendelian randomization study

**DOI:** 10.3389/fendo.2023.1133260

**Published:** 2023-07-27

**Authors:** Yangming Qu, Lanlan Chen, Shijie Guo, Ying Liu, Hui Wu

**Affiliations:** ^1^ Department of Neonatology, the First Hospital of Jilin University, Changchun, Jilin, China; ^2^ Department of Hepatobiliary and Pancreatic Surgery, the First Hospital of Jilin University, Changchun, Jilin, China

**Keywords:** uterine leiomyoma, risk factor, Mendelian randomization, influence, study

## Abstract

**Background and objective:**

Uterine leiomyoma is the most common benign tumor in females of reproductive age. However, its causes have never been fully understood. The objective of our study was to analyze the causal association between various factors and uterine leiomyoma using Mendelian randomization (MR).

**Methods:**

Genetic variables associated with risk factors were obtained from genome-wide association studies. Summary-level statistical data for uterine leiomyoma were obtained from FinnGen and the UK Biobank (UKB) consortium. We used inverse variance weighted, MR-Egger, and weighted median methods in univariate analysis. Multivariable MR analysis was used to identify independent risk factors. A fixed-effect model meta-analysis was used to combine the results of the FinnGen and UKB data.

**Results:**

In the FinnGen data, higher genetically predicted age at natural menopause, systolic blood pressure (SBP), diastolic blood pressure (DBP), and fasting insulin were associated with an increased risk of uterine leiomyoma, while higher age at menarche was associated with a reduced risk of uterine leiomyoma. Multivariable MR analysis of SBP and DBP showed that higher DBP might be an independent risk factor of uterine leiomyoma. In the UKB data, the results for age at natural menopause, SBP, DBP, and age at menarche were replicated. The result of the meta-analysis suggested that uterine leiomyoma could also be affected by polycystic ovary syndrome (PCOS), endometriosis, and 2-hour glucose level.

**Conclusion:**

Our MR study confirmed that earlier menstrual age, hypertension, obesity, and elevated 2-hour glucose post-challenge were risk factors for uterine leiomyoma, and the causal relationship between smoking and uterine leiomyoma was ruled out. In addition, later age of menopause and endometriosis were found to increase the risk of uterine leiomyoma, while PCOS was found to decrease the risk.

## 1 Introduction

Uterine leiomyomas (fibroids) are the most common benign tumor in females of reproductive age. Due to differences in race, diagnostic criteria, and study designs, epidemiological reports of uterine leiomyoma incidence vary widely, ranging from 4.5 to 68.6% (1). Since many uterine leiomyomas are asymptomatic and the histological incidence is more than twice as high as the clinical incidence, its true incidence may be underestimated in most studies (2). Johnson et al. (3) reported that the incidence of uterine leiomyoma increased with age, with a cumulative incidence of over 70% by menopause. In the United States, uterine leiomyoma accounts for 29% of gynecological hospitalizations among women aged 15-54 and 40-60% of all hysterectomies (4, 5).

Uterine leiomyoma has been shown to seriously impair woman’s quality of life and may lead to endometrial cancer (6). It can cause extensive or prolonged menstrual bleeding (leading to anemia, fatigue, and dysmenorrhea), abdominal swelling, painful intercourse, bladder or bowel dysfunction (leading to urinary incontinence or retention, pain, or constipation), and reproductive problems (such as impaired fertility, pregnancy complications, and miscarriage) (7, 8). If left untreated, it can even lead to death (9).

At present, the main treatment of uterine leiomyoma is hysterectomy, which is expensive and affects fertility. Nearly a quarter of women who have tried non-surgical treatment for fibroids choose to have surgery within a year (10). Many women opt for minimally invasive treatments to preserve their uterus, such as myomectomy, uterine artery embolization, and endometrial ablation. However, relapse is common after treatment (10). Therefore, it is necessary to clarify the risk factors of uterine leiomyoma for early prevention.

Several risk factors such as early age at menarche, early age at first birth, obesity, and hypertension have been established as increasing the risk of uterine leiomyoma (5, 11). However, due to the large number of undetected patients and the large bias of epidemiological data and risk factor evidence, its etiology is still far from being fully understood. In addition, some conflicting conclusions make it difficult to discover the true cause of uterine leiomyoma. For example, earlier studies have shown that smoking has a protective effect on fibroids (12, 13), while subsequent studies have shown that smoking increases the risk of uterine leiomyoma (14). One study showed that, among black women, those who self-reported PCOS had a 65% increased risk of fibroids compared with those who did not self-report PCOS (15). However, another study has shown that patients with PCOS had a lower risk of fibroids than women with normal ovaries (16). Additionally, some studies have found an inverse correlation between diabetes and uterine leiomyoma (13, 15, 17), and other researchers hypothesize that insulin stimulates fibroid growth (18, 19).

Therefore, whether there is a causal relationship between uterine leiomyoma and these factors still needs further analysis. Mendelian randomization (MR) is an emerging method of epidemiological causal inference, which uses genetic variation to determine the causal relationships between risk factors and outcomes. It relies on the natural random assortment of genetic variation during meiosis to distribute genetic variation randomly in a population, reducing bias caused by confounding or reverse causation (20). In our study, MR was used to explore the causal relationship between 20 risk factors and uterine leiomyomas. To our knowledge, this is the first MR study to examine the risk factors for uterine leiomyoma.

## 2 Methods

### 2.1 Summary statistics for risk factors

The summary statistics of anthropometric traits were from the GIANT (Genetic Investigation of Anthropometric Traits) consortium. For body mass index (BMI), the genome-wide association study (GWAS) included 234,069 Europeans and used sex, age, age squared, and principal components as covariates (21). For waist circumference, hip circumference, and waist-to-hip ratio, the GWAS included 210,088 Europeans and adjusted for age, age square, and study-specific covariates if necessary (22).

The summary statistics of DBP and SBP were obtained from the International Consortium for Blood Pressure, with 757,601 participants of European ancestry, and sex, age, and age squared were adjusted (23).

The summary statistics of serum 25-hydroxyvitamin D concentrations were from the SUNLIGHT consortium with 79,366 participants of European ancestry (24). The lead genetic variants of plasma vitamin C were derived from a GWAS meta-analysis of 52,018 Europeans from the Fenland study, the European Prospective Investigation into Cancer and Nutrition (EPIC)-InterAct study, the EPIC-Norfolk study, and the EPIC-CVD study (25).

In terms of smoking and drinking, the GWAS was conducted by the Sequencing Consortium of Alcohol and Nicotine use, which included 249,752 European participants for smoking and 335,394 European participants for drinking (26). Smoking was defined as the average number of cigarettes smoked per day, while drinking was the average number of alcoholic drinks consumed per week (including all types of alcohol). Age, sex, age-by-sex interaction, and the top 10 genetic principal components were used as covariates.

Three reproductive traits were involved in our study. The summary statistics of age at menarche (AAM) were from the largest meta-analysis of the ReproGen consortium, 23andMe, and the UK Biobank cumulatively including 329,345 women of European ancestry (27). The summary statistics of age at natural menopause (ANM) were from the ReproGen consortium with 69,360 European women (28). The summary statistics of age at first birth (AFB) were from a GWAS with 69,360 European individuals (29).

The summary statistics of PCOS were from a large-scale genome-wide meta-analysis with 10,074 PCOS cases and 103,164 controls of European ancestry (30). Cases were diagnosed with PCOS based on National Institutes of Health (NIH) or Rotterdam Criteria or by self-report, and age and BMI were used as covariables. The summary data for endometriosis included 17,045 endometriosis cases and 191,858 controls, 93% of whom were European (31).

The summary-level genetic data of T2D (Type 2 diabetes) were from the Diabetes Genetics Replication and Meta analysis consortium (32). A total of 74,124 T2D cases and 824,006 controls of European ancestry from 32 GWAS (with and without adjustment for BMI) were included.

The GWAS summary statistics of glycemic traits, including fasting glucose, fasting insulin, glycated hemoglobin (HbA1c), and 2-hour glucose post-challenge in an oral glucose tolerance test, were obtained from MAGIC (Meta-Analyses of Glucose and Insulin-related traits Consortium) (33). The GWAS included 281,416 participants, 70% of whom were of European ancestry. Our study used only European summary statistics and adjusted for covariates specific to the study.

### 2.2 GWAS summary statistics of uterine leiomyoma from the FinnGen and UKB consortia data sets

The GWAS summary statistics of uterine leiomyoma were obtained from FinnGen (https://r4.finngen.fi/) and the UKB. In the FinnGen data, the GWAS included 18,060 cases and 105,519 controls of European ancestry. The GWAS from the UKB included 4,351 cases and 332,848 controls and was conducted by the Neale Lab (http://www.nealelab.is/uk-biobank). Uterine leiomyoma is defined as ICD (International Classification of Diseases) 10: D25. When assessing causality, the FinnGen GWAS was used as the discovery set and the UKB GWAS as the validation set, considering that the FinnGen data had a higher proportion of cases. The main design of this study is shown in **Supplementary Figure 1**.

### 2.3 Ethics and consent statement

Specific ethical and consent statements for each GWAS in this study can be found in the original GWAS publications. The FinnGen Biobank GWAS was approved by the FinnGen Steering Committee. The Neale Lab received approval to conduct the GWAS from the Ethics Advisory Committee of the UKB. All of these data are de-identified, freely downloadable, and can be used without restriction.

### 2.4 Statistical analysis

We used a two-sample Mendelian randomization analysis to explore the potential causal relationship between 20 risk factors and uterine leiomyoma. Single nucleotide polymorphism (SNPs) with genome-wide significance (P < 5 × 10^-8^) and minor allele frequency >0.01 were included. Then, these SNPs were clumped based on the linkage disequilibrium r^2^< 0.01. The power of each SNP was assessed using F statistics (34) (F = beta^2^/se^2^), and the general F statistics of each exposure were also calculated. SNPs with weak statistical power were deleted (F statistics< 10).

We used inverse variance weighted (IVW) analysis as the primary statistical method. Although this method assumes that there is no heterogeneity between genetic variants (potentially due to pleiotropy), it has the strongest power to detect associations (35). In addition, we used two sensitivity analyses methods, including the MR-Egger (36) and weighted median (37) methods, as supplements to IVW. MR-Egger intercept and MR-PRESSO (38) methods were used to detect horizontal pleiotropy, and Cochran’s Q statistic was used to assess the heterogeneity. When there were outliers, the MR-PRESSO-corrected results would be reported in the main results. If heterogeneity still exists, the median based estimation was used as primary analysis. A false discovery rate (FDR) was used to adjust for multiple testing. In multivariable MR (MVMR) analysis, the IVW model was also the main method and the MR-Egger method was the complementary method.

A fixed-effects model meta-analysis was used to combine the results of the training set and verification set. All statistical analyses were performed using R software 4.1.2 (https://www.r-project.org/). The IVW, MR-Egger and weighted median methods were performed using the R packages “Two Sample MR” and “Mendelian Randomization”. The MVMR was performed using the R packages “Mendelian Randomization” and “MVMR”. P<0.05 was used as significance threshold. The mRnd was used to calculate the statistical power (39) for MR (https://cnsgenomics.shinyapps.io/mRnd/).

## 3 Results

### 3.1 Summary characteristics of risk factors

The number of SNPs ranged from 6 to 821, explaining 0.15% to 7.01% of the variance. The F statistics of each SNP and exposure were greater than 10, indicating that all instrumental variables had sufficient validity (**Table 1**).

**Table 1:** Summary characteristics of risk factors.

Exposure	Data source	NSNP	Unit	Sample	R^2^(%)	F	PMID
BMI	GIANT consortium	92	SD	234,069	2.39	62.27	25673413
Serum 25-hydroxyvitamin D concentrations	SUNLIGHT consortium	6	SD	79,366	0.8	106.67	31100827
Drinking	Sequencing Consortium of Alcohol and Nicotine use	39	SD	335,394	0.84	72.84	30643251
PCOS	A large-scale genome-wide meta-analysis	14	logOR	113,238	0.50	40.64	30566500
Endometriosis	GWAS	14	logOR	208,903	0.26	38.89	28537267
Smoking	Sequencing Consortium of Alcohol and Nicotine use	28	SD	249,752	1.04	93.73	30643251
Age at menarche	ReproGen consortium	321	SD	329,345	6.29	68.80	28436984
2-hour glucose	MAGIC	14	SD	281,416	0.31	62.50	34059833
Fasting glucose	MAGIC	69	SD	281,416	2.74	114.87	34059833
Fasting insulin	MAGIC	36	SD	281,416	0.70	55.10	34059833
HbA1c	MAGIC	78	SD	281,416	2.84	105.43	34059833
Age at natural menopause	ReproGen consortium	48	SD	69,360	4.37	65.99	26414677
Age at first birth	GWAS	10	SD	251,151	0.15	37.73	27798627
T2D	Diabetes Genetics Replication and Meta-analysis consortium	248	logOR	898,130	1.78	65.61	30297969
SBP	International Consortium for Blood Pressure	776	SD	757,601	6.49	67.69	30224653
DBP	International Consortium for Blood Pressure	821	SD	757,601	7.01	69.49	30224653
Circulating vitamin C concentration	A GWAS meta-analysis	10	SD	52,018	1.72	91.02	33203707
Waist-to-hip ratio	GIANT consortium	34	SD	210,088	0.76	47.31	25673412
Waist circumference	GIANT consortium	45	SD	210,088	1.26	59.56	25673412
Hip circumference	GIANT consortium	55	SD	210,088	1.42	55.01	25673412

### 3.2 Discovery results of uterine leiomyoma in the FinnGen consortium data set

In the FinnGen data set, a higher genetically predicted age at natural menopause (OR=1.0864 per standard deviation of age at natural menopause increase, 95%CI=1.0429-1.1317,*P*=6.97×10^-5^), SBP (OR=1.0073 per standard deviation of SBP increase, 95%CI=1.0026-1.0120,*P*=2.30×10^-3^), DBP (OR=1.0118 per standard deviation of DBP increase, 95%CI=1.0040-1.0197,*P*=3.09×10^-3^), and fasting insulin (OR=1.7342 per standard deviation of fasting insulin increase, 95%CI=1.1455-2.6253, *P*=9.25×10^-3^) were associated with an increased risk of uterine leiomyoma, while a genetically predicted higher age at menarche (OR=0.8435 per standard deviation of age of menarche increase, 95%CI=0.7999-0.8894, *P*=3.27×10^-10^) was associated with a reduced risk of uterine leiomyoma (FDR<0.05). T2D, endometriosis, and BMI showed a positive association with uterine leiomyoma risk (FDR> 0.05 and IVW *P*< 0.05). Both SBP and DBP are indicators of blood pressure, and some SNPs may be associated with both SBP and DBP. Therefore, we used multivariable MR to adjust the results of SBP and DBP. Multivariable MR analysis of SBP and DBP showed that a higher DBP might be an independent risk factor of uterine leiomyoma (adjusted OR=1.0309, 95%CI=1.0091-1.0533, *P*=5.34×10^-3^), while SBP was not significant (adjusted OR=0.9913, 95%CI=0.9787-1.0041, *P*=0.184).

The results of heterogeneity, pleiotropy, weighted median, and MR-Egger are shown in **Table 2**. There was heterogeneity in age at natural menopause, SBP, DBP, fasting insulin, age of menarche, T2D, and endometriosis, and they all showed MR-PRESSO-corrected results if outliers were detected. No horizontal pleiotropy was found. For some robust MR estimators, such as endometriosis, age at menarche, age at natural menopause, fasting insulin, T2D, and SBP, their IVW results were supported by MR-Egger or weighted median. However, the IVW results of waist-to-hip ratio and DBP were not supported by MR-Egger or weighted median, which may be related to the presence of horizontal pleiotropy and the detected outliers. The statistical power for the FinnGen outcome ranged from 96% to 100%.

**Table 2:** Two-sample Mendelian randomization estimates of MR-Egger and weighted median methods.

	NSNP	MR-Egger					Weighted median				*P*_heterogenelty_	*P*_pleiotropy_
		OR	95%LCI	95%UCI	*p*		OR	95%LCI	95%UCI	*p*		
FinnGen												
BMI	91	1.2241	0.9059	1.6539		0.191	1.2042	1.0064	1.4409	0.049	1.114	0.589
Waist-to-hip ratio	33	1.5922	0.6209	4.0826		0.340	1.2690	0.9468	1.7007	0.111	0.005	0.503
Waist circumference	45	0.9948	0.5621	1.7607		0.986	1.1309	0.9001	1.4209	0.291	0.307	0.689
Hip circumference	54	1.3795	0.8947	2.1270		0.151	1.1408	0.9363	1.3900	0.191	0.076	0.210
Serum 25-hydroxyvitamin D concentrations	6	1.6075	0.9683	2.6684		0.140	1.3320	0.9813	1.8081	0.066	0.420	0.301
Circulating vitamin C concentration	10	1.0356	0.8117	1.3213		0.785	0.9769	0.8169	1.1683	0.798	0.647	0.585
Drinking	39	0.3618	0.1484	0.8818		0.031	0.4423	0.2678	0.7308	0.001	0.003	0.094
Smoking	28	0.9393	0.8054	1.0956		0.433	0.9348	0.8267	1.0570	0.282	0.937	0.757
PCOS	13	0.6201	0.3372	1.1406		0.153	0.8898	0.8089	0.9788	0.016	<0.001	0.261
Endometriosis	14	5.0109	0.8617	29.141		0.098	1.1982	1.0337	1.3889	0.016	<0.001	0.189
Age at menarche	303	0.8870	0.7682	1.0243		0.103	0.8584	0.7904	0.9324	<0.001	<0.001	0.461
Age at natural menopause	43	1.2002	1.0752	1.3397		0.002	1.1337	1.0906	1.1784	<0.001	<0.001	0.064
Age at first birth	10	0.7951	0.3458	1.8282		0.604	1.0355	0.9139	1.1732	0.584	0.886	0.577
2-hour glucose	13	0.6588	0.3459	1.2547		0.230	0.9582	0.8047	1.1409	0.631	<0.001	0.138
Fasting glucose	65	1.1888	0.7707	1.8337		0.437	1.1895	0.9260	1.5281	0.174	<0.001	0.347
Fasting insulin	36	1.6367	0.4093	6.5449		0.491	1.7667	1.0794	2.8915	0.024	<0.001	0.932
HbA1c	74	1.2990	0.7970	2.1172		0.297	1.0847	0.7648	1.5386	0.648	0.079	0.339
T2D	230	0.9826	0.9149	1.0553		0.631	1.0539	1.0009	1.1096	0.046	<0.001	0.054
SBP	741	1.0139	1.0016	1.0263		0.027	1.0051	0.9989	1.0113	0.105	<0.001	0.255
DBP	776	1.0061	0.9866	1.0260		0.544	1.0060	0.9953	1.0167	0.275	<0.001	0.533
UKB												
BMI	91	0.9990	0.9931	1.0049		0.736	1.0024	0.9988	1.0059	0.197	0.633	0.501
Waist-to-hip ratio	33	0.9964	0.9754	1.0178		0.741	1.0047	0.9982	1.0113	0.153	0.010	0.505
Waist circumference	44	1.0014	0.9896	1.0133		0.817	1.0008	0.9958	1.0058	0.760	0.605	0.910
Hip circumference	54	1.0029	0.9946	1.0113		0.498	1.0030	0.9988	1.0072	0.152	0.483	0.651
Serum 25-hydroxyvitamin D concentrations	6	0.9973	0.9875	1.0072		0.621	0.9972	0.9910	1.0035	0.384	0.925	0.966
Circulating vitamin C concentration	10	0.9975	0.9921	1.0029		0.383	0.9989	0.9953	1.0024	0.531	0.126	0.316
Smoking	28	0.9990	0.9957	1.0024		0.580	1.0000	0.9972	1.0030	0.972	0.497	0.935
Drinking	38	0.9991	0.9879	1.0104		0.877	1.0010	0.9923	1.0099	0.812	0.037	0.858
PCOS	12	0.9901	0.9810	0.9995		0.066	0.9979	0.9957	1.0000	0.052	0.047	0.153
Endometriosis	14	1.0142	0.9938	1.0350		0.200	1.0041	1.0013	1.0071	0.005	<0.001	0.333
Age at menarche	306	0.9974	0.9958	0.9991		0.075	0.9983	0.9966	0.9999	0.049	0.031	0.686
Age at natural menopause	45	1.0016	1.0000	1.0033		0.058	1.0011	1.0004	1.0019	0.003	<0.001	0.677
Age at first birth	10	0.9987	0.9812	1.0166		0.891	1.0023	0.9996	1.0051	0.096	0.583	0.788
2-hour glucose	14	0.9994	0.9907	1.0082		0.897	1.0014	0.9981	1.0047	0.416	0.046	0.375
Fasting glucose	67	1.0011	0.9936	1.0086		0.781	1.0029	0.9975	1.0083	0.299	0.065	0.542
Fasting insulin	35	0.9907	0.9671	1.0148		0.450	1.0002	0.9905	1.0100	0.967	0.097	0.458
HbA1c	75	1.0013	0.9923	1.0103		0.782	0.9983	0.9913	1.0054	0.645	0.136	0.830
T2D	236	1.0003	0.9990	1.0017		0.639	1.0003	0.9991	1.0015	0.665	0.005	0.937
SBP	757	1.0001	0.9999	1.0003		0.330	1.0000	0.9999	1.0001	0.448	0.038	0.968
DBP	794	1.0000	0.9996	1.0004		0.931	1.0002	1.0001	1.0004	0.045	<0.001	0.279

### 3.3 Validation results of uterine leiomyoma in the UKB consortium data set

**Figure 1:**
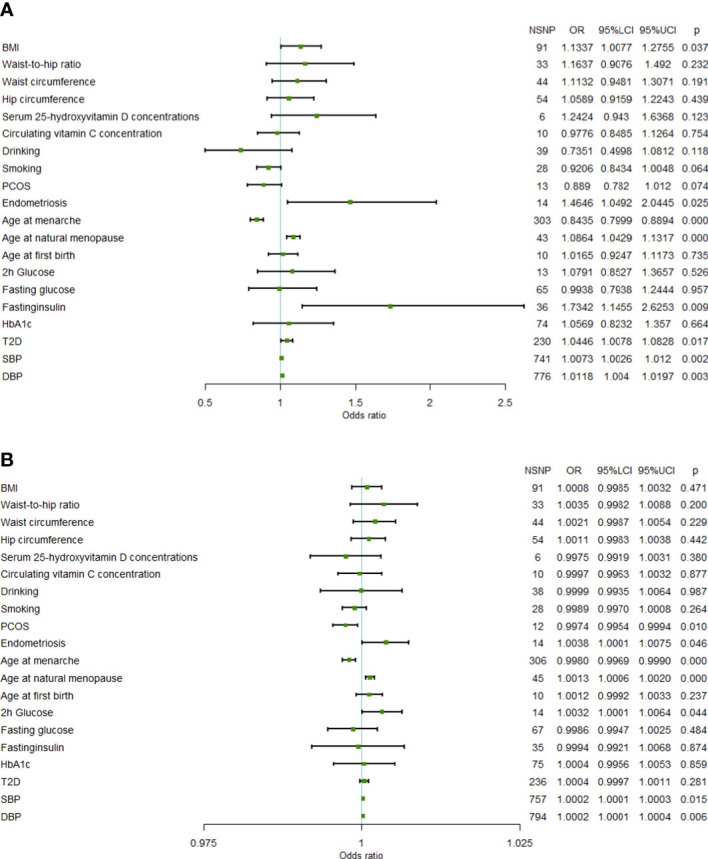
Forest plot of Mendelian randomization results. **(A)** Mendelian randomization results in the FinnGen data set. **(B)** Mendelian randomization results in the UKB data set (95%LCI, lower limit of 95% CI; 95%UCI, upper limit of 95% CI; BMI, body mass index; PCOS, polycystic ovary syndrome; 2h glucose, 2-hour glucose after oral glucose tolerance test; HbA1c, glycated hemoglobin; T2D, Type 2 diabetes; SBP, systolic blood pressure; DBP, diastolic blood pressure; NSNP, number of single nucleotide polymorphisms).

In the validation set, the MR results of SBP, DBP, age at menarche, and age at natural menopause were consistent with the training set. A higher age at natural menopause, SBP, and DBP were associated with an increased risk of uterine leiomyoma, while a higher age of menarche was associated with a reduced risk of uterine leiomyoma (**Figure 1**). No horizontal pleiotropy was found for these risk factors. After removing outliers, the odds of uterine leiomyoma increased per 1-SD increase in SBP (OR=1.0002, 95%CI=1.0001-1.0003, *P*=1.01×10^-4^), age at natural menopause (OR=1.0013, 95%CI=1.0006-1.0020, P=2.08×10^-4^), and DBP (OR=1.0002, 95%CI=1.0001-1.0004, *P*=8.95×10^-3^). Moreover, 1-SD increase in age at menarche (OR=0.9980, 95%CI=0.9969-0.9990, *P*=1.73×10^-4^) was associated with a reduced risk of uterine leiomyoma.

In addition, the results of the validation set showed that genetic liability to PCOS (OR=0.9974, 95%CI=0.9954-0.9994, *P*=1.09×10^-2^), 2-hour glucose level (OR=1.0032, 95%CI=1.0001-1.0064, *P*=4.57×10^-2^), and endometriosis (OR=1.0038, 95%CI=1.0001-1.0075, *P*=4.34×10^-2^) were also influential factors for uterine fibroids.

It is worth noting that the statistical power of the UKB results was not sufficient (<50%). The reason may be that UKB data set has fewer cases than the FinnGen data set, resulting in lower statistical power.

### 3.4 Combined result of uterine leiomyoma from meta-analysis

**Figure 2:**
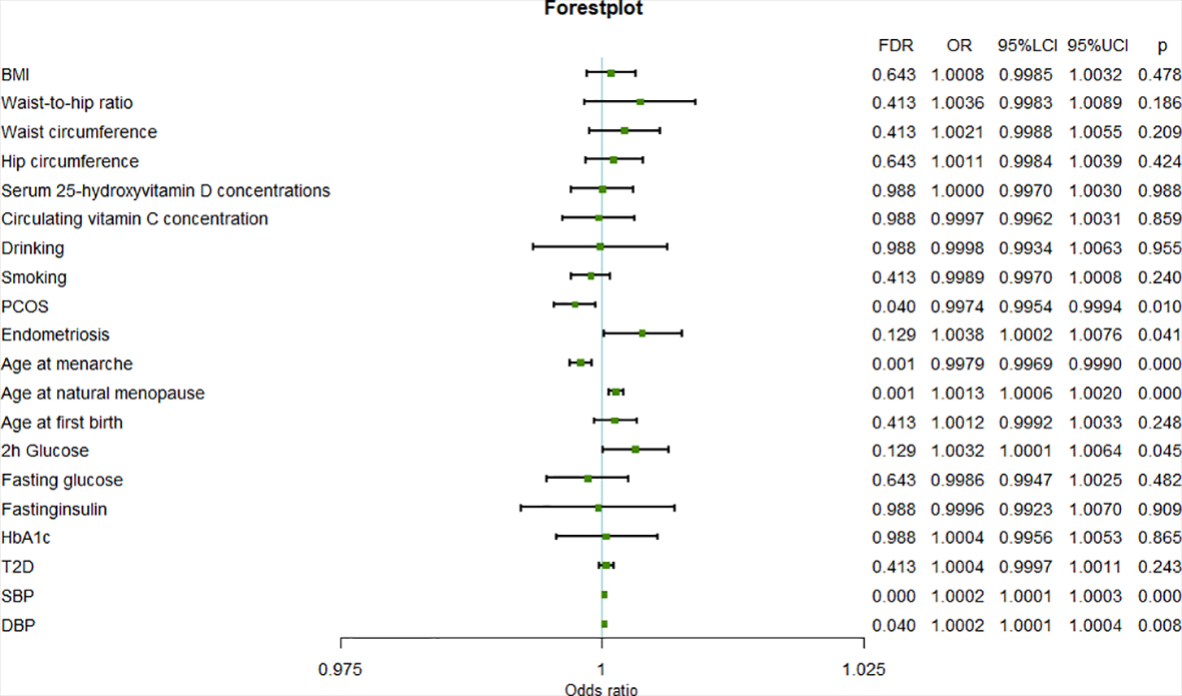
Forest plot of the results from meta-analysis.

The results of the meta-analysis further confirmed the previous findings that a higher age at natural menopause (OR=1.0013, 95%CI=1.0006-1.0020, *P*=2.94×10^-4^), SBP (OR=1.0002, 95%CI=1.0001-1.0003, *P*=1.01×10^-4^), and DBP (OR=1.0002, 95%CI=1.0001-1.0003,*P*=8.95×10^-3^) are risk factors for uterine fibroids, and a higher age at menarche (OR=0.9979, 95%CI=0.9969-0.9990, *P*=1.02×10^-4^) is a protective factor for uterine leiomyoma (**Figure 2**).

In addition, the results of meta-analysis suggested that uterine leiomyoma may also be affected by PCOS (OR=0.9974, 95%CI=0.9954-0.9994, *P*=1.09×10^-2^), endometriosis (OR=1.0038, 95%CI=1.0002-1.0076, *P*=4.33×10^-2^), and 2-hour glucose levels (OR=1.0032, 95%CI=1.0001-1.0064, *P*=4.57×10^-2^). In fact, PCOS and endometriosis were also significant in the FinnGen results, although they failed to pass FDR correction. Therefore, the results of analysis of the FinnGen and UKB data sets and the meta-analysis of PCOS and endometriosis were consistent. The difference between the results of the FinnGen and UKB data sets for 2-hour glucose level may be related to the different number of SNPs in the instrumental variables.

## 4 Discussion

Our MR study found that a genetically predicted higher age at natural menopause, SBP, DBP, endometriosis, and elevated 2-hour glucose level were risk factors for uterine leiomyoma, and a higher age at menarche and PCOS were protective factors for uterine leiomyoma.

An earlier age at menarche is thought to be associated with an increased risk of uterine leiomyoma (40, 41), and this finding is further supported by our study. Women with an earlier age at menarche had higher levels of estradiol and estrone and lower levels of sex hormone-binding globulin in their hormonal milieu than women with a later age at menarche (42, 43). Fibroids were found to have more estrogen receptors, lower estradiol metabolism, and a stronger transcriptional response to estrogen than myometrium (44). Therefore, higher estrogen and progesterone levels may increase the risk of fibroids. Animal models have also confirmed that hormonal stimulation can increase tumor proliferation and decrease apoptosis (45). There are few studies on the effect of menopausal age on uterine leiomyoma risk. Our results suggest that a later age at menopause is associated with an increased risk of uterine leiomyoma. A prospective study of female teachers also found a reduced risk of fibroids in postmenopausal women compared to premenopausal women (13). The National Institute of Environmental Health Sciences (NIEHS) Fibroid Growth Study found that the growth rate of uterine leiomyoma in white women was related to age (46). Rapid growth of uterine leiomyoma after the age of 30, especially in premenopause, is consistent with age-related changes in estrogen and progesterone (47). Therefore, the effect of menopausal age on fibroids may also be related to hormone levels. In addition, mitotic activity in the myometrium is greatest during the luteal phase of the menstrual cycle, and prolonged exposure to the menstrual cycle may increase the risk of uterine leiomyoma (48). This also suggests that earlier menstruation age and later menopause age can increase the risk of uterine leiomyoma.

Several studies have found a significant positive association between hypertension and uterine leiomyoma, but most of these studies are retrospective studies, cross-sectional studies, or prospective studies with small sample sizes and have not successfully established a causal association (49–51). Our results suggest that higher SBP and DBP are causally associated with an increased risk of uterine leiomyoma. During the onset of hypertension, angiotensin is hydrolyzed to angiotensin I, which is then converted to angiotensin II by the angiotensin converting enzyme (ACE) (52). Angiotensin II has been reported to significantly increase the number of uterine leiomyoma cells in a dose-dependent manner (53). Hsieh et al. found that mutations in angiotensin-converting enzyme activation genes were significantly associated with leiomyoma susceptibility (54). A recent study reported a 31.8% reduction in clinically diagnosed uterine leiomyoma in hypertensive adult women who had previously used angiotensin-converting enzyme inhibitors (ACEis) compared with those who had not used ACEis (55). Therefore, hypertension may cause uterine leiomyoma through production of angiotensin II. In addition, hypertension can induce fibroid proliferation and fibrogenesis by inducing smooth muscle cell injury through mechanical shear stress, which may also lead to uterine leiomyoma (56).

The relationship between diabetes mellitus and uterine leiomyoma has been controversial for many years. On the one hand, some observational studies have found a lower incidence of fibroids in diabetic patients and hypothesized that diabetes may inhibit tumor development by causing vascular dysfunction (57). On the other hand, diabetes is often accompanied by obesity and hypertension, which may increase the risk of uterine leiomyoma. Since it is not possible to assess the relationship between diabetes and fibroids in untreated diabetic populations, observational estimates may capture both the effect of disease and treatment effects on fibroids. A study that indirectly examined the relationship between diabetes and treatment found that the protective effects of diabetes was only present in diabetic patients receiving the drug (15). Elevated 2-hour glucose post-challenge, an indicator of diabetes, was found in our study to be associated with an increased risk of uterine leiomyoma. However, fasting glucose, another indicator of diabetes, was not causally associated with uterine leiomyoma in either the FinnGen or the UKB data sets. In addition, elevated fasting insulin levels and type 2 diabetes were found to be associated with an increased risk of uterine leiomyoma in the FinnGen data, but this association was not found in the UKB data. Due to the low power of the UKB results, we were unable to determine the relationship between diabetes and uterine leiomyoma. To be sure, the risk of uterine leiomyoma should be considered when 2-hour glucose post-challenge is elevated.

Endometriosis polycystic ovary syndrome (PCOS) and uterine leiomyoma are common non-cancerous gynecological diseases in women. Our study found that endometriosis was associated with an increased risk of uterine leiomyoma, while PCOS was associated with a reduced risk of uterine leiomyoma. Although there is no direct evidence that endometriosis is a influence factor of uterine leiomyomas, Uimari et al. found that 20% of patients with symptomatic fibroids had endometriosis, and 26% of patients with symptomatic endometriosis had fibroids (58). Hemmings et al. (59) also reported that patients with endometriosis were more likely to develop uterine leiomyoma than patients without endometriosis. Physiologically, endometriosis tissues have been shown to express aromatase and produce estrogen independently of the ovary (60), which is a major cause of uterine leiomyoma. Therefore, aromatase inhibitors for endometriosis may reduce the risk of uterine leiomyoma. There are few studies on the relationship between PCOS and uterine leiomyoma, and existing studies are controversial (15, 16). Our study found that PCOS was associated with a reduced risk of uterine leiomyoma. Our results are consistent with a large later study using data from PPCOS I (National Institute of Child Health and Human Development Cooperative Reproductive Medicine Network Pregnancy in Polycystic Ovary Syndrome I), PPCOS II (Pregnancy in Polycystic Ovary Syndrome II), and AMIGOS (Assessing Multiple Intrauterine Gestations from Ovarian Stimulation) and found PCOS patients had a reduced risk of uterine leiomyoma compared to unexplained infertility patients (61). The results of this large study using ultrasound diagnosis were more reliable than those of a small sample using self-reported data. PCOS patients are anovulatory and have limited exposure of myometrium to progesterone, which has been shown to stimulate leiomyoma growth through a group of key genes that regulate apoptosis and proliferation, and may be the cause of this association (62–64).

Obesity has been consistently recognized as a risk factor for uterine leiomyoma. Given the insufficient power of the UKB database and the fact that a recent Mendelian randomization study (65) using different instrumental variables found that a higher BMI slightly increased the risk of uterine fibroids in the UKB data set, we concluded that obesity is unquestionably a risk factor for uterine leiomyomas.

Several studies have reported a negative association between age at first birth and uterine leiomyoma risk (40, 41, 66), while our study found no such association. Pregnancy may lead to decreased estrogen receptor levels in myometrium (67). Postpartum reduction of collagen content and smooth muscle cytoplasm can eliminate or shrink uterine leiomyoma (68). In addition, the vascular distribution of uterine leiomyoma is different from that of the myometrium, and delivery ischemia and uterine remodeling can give priority to the elimination of uterine leiomyoma (69, 70). But the relationship between age at first birth and uterine leiomyoma may be non-linear. Donnaet al. (71) reported that the effect of age at first birth on uterine leiomyoma was not linear, and mid-reproductive (25-29 years) delivery appeared to be most protective against fibroids development. Larger fibroids are more common in women over the age of 40. If women give birth at a young age, the disease may not develop. But there may be no benefit if the first pregnancy is too late, as some tumors may have grown too large. Our study was unable to determine whether there is a non-linear relationship between age and uterine leiomyoma risk, so more research is needed.

There are some advantages to our study: (1) This was a Mendelian randomized study that could find causal associations. (2) Our study found that some previously unknown factors, such as uterine leiomyoma and menopausal age, were associated with uterine leiomyoma. In addition, the influence of previously controversial factors such as PCOS, smoking, and diabetes on uterine leiomyoma were identified. And (3), participants in all GWAS studies were of predominantly European ancestry, with less racial bias. Discovery data sets, validation sets, and meta-analysis were used to increase the reliability of the results. Our research also has some shortcomings: (1) The influence of pleiotropy in the MR design, including horizontal pleiotropic and vertical pleiotropic; we used two sensitivity analysis methods to detect pluripotency, including MR-Egger intercept and MR-PRESSO, in the hope of minimizing bias. (2) The power of the results verified in the UKB data set was lower, resulting in several factors that were found to be significant in the FinnGen data set but not in the UKB. (3) The fact that the GWAS studies were mainly Europeans may have influenced the extrapolation of the results. In addition, the non-linear relationships could not be detected in this study. And (4), the genetic instruments were variants identified through GWAS analyses with p-values < 5x10^-8^. As a result, the estimates of these genetic effects tend to be upwardly biased due to a phenomenon known as the “winner’s curse”.

In conclusion, our MR study confirmed that earlier menstrual age, hypertension, obesity, and elevated 2-hour glucose post-challenge were risk factors for uterine leiomyoma, and ruled out the causal relationship between smoking and uterine leiomyoma. In addition, a later age of menopause and endometriosis were found to increase the risk of uterine leiomyoma, while PCOS was found to decrease the risk.

## Data availability statement

The datasets presented in this study can be found in online repositories. The names of the repository/repositories and accession number(s) can be found below: ReproGen: (http://www.reprogen.org/); GIANT: (http://portals.broadinstitute.org/collaboration/giant/index.php/GIANT_consortium_data_files); MAGIC: (https://magicinvestigators.org/downloads/); GLGC: (http://lipidgenetics.org/#data-downloads-title); UKB: (http://www.nealelab.is/uk-biobank); FinnGen: (https://r4.finngen.fi/).

## Ethics statement

Ethical review and approval was not required for the study on human participants in accordance with the local legislation and institutional requirements. The patients/participants provided their written informed consent to participate in this study. All the data used were from the GWAS studies, specific ethical and consent statements for each GWAS in this study can be found in the original GWAS publications.

## Author contributions

HW and YQ conceptualized and designed the study, drafted the initial manuscript, and reviewed and revised the manuscript. YQ and LC collected the data and carried out the initial analyses. SG and YL reviewed and revised the manuscript. All authors approved the final manuscript as submitted and agree to be accountable for all aspects of the work.

## Supplementary material

Supplementary Figure 1 Main design of this study.
