# Supplementary figures and images for: Genetic liability to multiple factors and uterine leiomyoma risk: a Mendelian randomization study
Source: Front Endocrinol (Lausanne). 2023 Jul 27;14:1133260. doi: 10.3389/fendo.2023.1133260 (PMC10415162; doi:10.3389/fendo.2023.1133260)

Supplementary Figure 1. Main design of this study


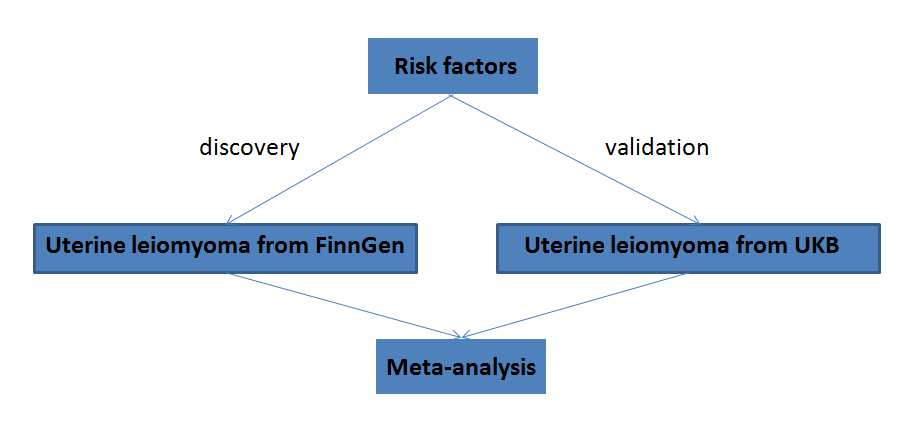

Supplement: Supplementary Figure 1 — Main design of this study. [file DataSheet_1.docx]
